# Time-averaged simulated microgravity (taSMG) inhibits proliferation of lymphoma cells, L-540 and HDLM-2, using a 3D clinostat

**DOI:** 10.1186/s12938-017-0337-8

**Published:** 2017-04-20

**Authors:** Yoon Jae Kim, Ae Jin Jeong, Myungjoon Kim, Chiwon Lee, Sang-Kyu Ye, Sungwan Kim

**Affiliations:** 1 Interdisciplinary Program for Bioengineering, Graduate School, Seoul National University, Seoul, 08826 South Korea; 2 Department of Pharmacology and Biomedical Sciences, Seoul National University College of Medicine, Seoul, 03080 Republic of Korea; 3 Department of Biomedical Sciences, Seoul National University College of Medicine, Seoul, 03080 Republic of Korea; 4 Ischemic/Hypoxic Disease Institute, Seoul National University College of Medicine, Seoul, 03080 Republic of Korea; 5 Biomedical Science Project (BK21PLUS), Seoul National University College of Medicine, Seoul, 03080 Republic of Korea; 6 Neuro-Immune Information Storage Network Research Center, Seoul National University College of Medicine, Seoul, 03080 Republic of Korea; 7 Institute of Medical and Biological Engineering, Medical Research Center, Seoul National University, Seoul, 03080 South Korea; 8 Department of Biomedical Engineering, Seoul National University College of Medicine, Seoul, 03080 South Korea

**Keywords:** Microgravity, Lymphoma, 3D clinostat, Dermal fibroblast

## Abstract

**Background:**

Gravity is omnipresent on Earth; however, humans in space, such as astronauts at the International Space Station, experience microgravity. Long-term exposure to microgravity is considered to elicit physiological changes, such as muscle atrophy, in the human body. In addition, certain types of cancer cells demonstrate inhibited proliferation under condition of time-averaged simulated microgravity (taSMG). However, the response of human Hodgkin’s lymphoma cancer cells to reduced gravity, and the associated physiological changes in these cells, have not been elucidated.

**Methods:**

In this study, the proliferation of human Hodgkin’s lymphoma cancer cells (L-540 and HDLM-2) under taSMG condition (<10^−3^ G, 1 G is defined as 9.8 m/s^2^) was studied using a 3D clinostat. Normal human dermal fibroblast (HDF) was proliferated in the same condition as a control group. For the development of 3D clinostat, two motors were used to actuate the frames. Electrical wires for power supply and communication were connected via slip ring. For symmetrical path of gravitational vector, optimal angular velocities of the motors were found using simulation results. Under the condition of taSMG implemented by the 3D clinostat, proliferation of the cells was observed for 3 days.

**Results:**

The results indicated that proliferation of these cancer cells was significantly (*p* < 0.0005) inhibited under taSMG, whereas proliferation of normal HDF cells was not affected.

**Conclusions:**

Findings in this study could be significantly valuable in developing novel strategies for selective killing of cancer cells such as lymphoma.

## Background

Gravity is an omnipresent force on Earth, under whose constant influence all living organisms have evolved. Thus, a study on the effects of microgravity on living organisms is a fascinating area of research. Since the 1960s, hundreds of astronauts that have spent time in space have shown various unpredictable physiological changes such as muscle atrophy, bone demineralization, immune dysregulation, and abnormal cellular functions [1–8]. These findings strongly indicate that the human body experiences physiological changes under microgravity. However, this is extremely challenging to study in vivo as microgravity is difficult to simulate; as such, long-term exposure to microgravity (10^−4^ G, 1 G is defined as 9.8 m/s^2^) may be experienced in space. Therefore, an alternative strategy, termed time-averaged simulated microgravity (taSMG), has been proposed, wherein a continuous change in the direction of gravity enables simulation of the effect of microgravity on cells. Previous studies have demonstrated that certain types of cells, such as leukocytes (human leukemic myelomonocytic cell line U937) [9, 10] and T lymphocytes [11–13], show similar results when exposed to real microgravity and taSMG. Accordingly, clinostats have been utilized to provide taSMG as an alternative of real microgravity condition despite physically different situation in which they are under.

On Earth, a real microgravity environment may be created by a free fall from a drop tower. However, the duration of microgravity obtained by these methods is generally too short for cell cultivation [14]. Thus, clinostat, which provides a long-term taSMG environment, has been applied in studies of the effects of microgravity on cells. Various types of clinostat have been proposed to provide efficient microgravity; one of the initial structures of clinostat was introduced by the German botanist Julius von Sachs in 1879 [15]. He constructed a 2D clinostat, which rotates along horizontal axis for microgravity simulation. Although 2D clinostats provide a practical method for simulation of microgravity, they are associated with unwanted effects [16]. Therefore, the idea of a 3D rotation came about in order to more closely simulate a microgravity environment. The 3D clinostat, so called random positioning machine [17, 18], consists of two perpendicular frames that rotate independently, and provides continuous rotation with two axes, enabling cells within the clinostat to experience taSMG. One of the first brief reports on a 3D clinostat was provided by the Italian scientist Aristide Scano in 1963 [19]. A paper reported that 3D clinostat can demonstrate improved simulation for real microgravity relative to the classical 2D clinostat [20] even though it can’t be generalized because 2-D clinostat especially running in the fast angular velocity provides a good simulation of microgravity and can be recommended for most biological organisms studied [17].

3D clinostats with improved algorithms and hardware have been developed by previous studies [21–23]. In terms of algorithm, the classical mode, which drives only one of the two frames with a constant speed, has been conventionally used. In the subsequent step, two perpendicular axes are rotated at a constant speed to provide 3D clinostat motion. However, these algorithms do not guarantee symmetrical coverage of all orientations [21]. By using random angular velocities for two axes, an unpredictable and symmetrical path of gravitational vectors was implemented [21]. Regarding the hardware, improved equipment is necessary for accuracy and convenience. A system for the control of the actuator on the platform could be operated while the clinostat is running [21], enabling valves, pumps, and motors inside the clinostat to be controlled by external Windows based programs. Communication and power were provided via the slip rings. Furthermore, temperature and CO_2_ controllable clinostats were developed respectively [21, 22], as environmental conditions, such as temperature, humidity and CO_2_ concentration are important factors. For a similar purpose, incubator embedded clinostats were developed [21]. The development of microscopy embedded clinostats, for real-time observation of experiments during the operation of clinostat, were also reported previously [23].

In recent years, taSMG exhibited potential to suppress migration and proliferation of various types of cancer cells found in brain, liver, breast, and lung [24–28]. Nevertheless, the cellular responses and physiological effects, in terms of proliferation of cancer cells in taSMG, remain to be explored. In the present study, we sought to determine whether taSMG is capable of inhibiting the proliferation of tumor cells and normal cells in a 3D clinostat. The effects of taSMG on the growth of human lymphoma cells have been investigated. Human lymphoma cell lines, L-540 and HDLM-2, were cultivated in the 3D clinostat with constant angular velocities. The angular velocities used were determined via an optimization process to provide evenly distributed gravity.

## Methods

### 3D clinostat development

**Fig. 1:**
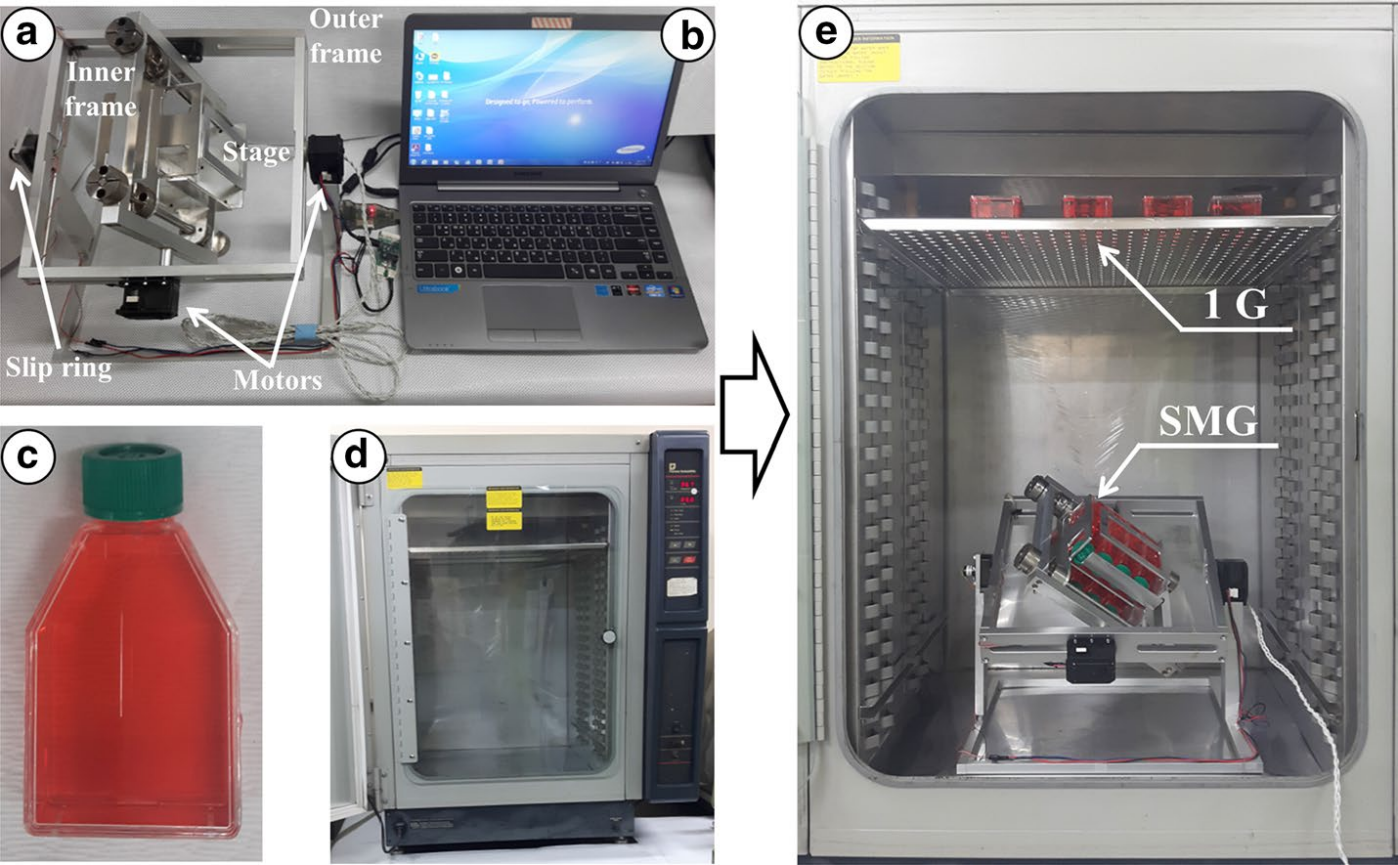
**a** Three dimensional clinostat hardware. The hardware consists of two frames, outer frame and inner frame, with perpendicular rotational axes. The power and communication wires are connected via the slip ring. **b** PC for control, **c** 25 cm^2^ flask for cell proliferation, **d** incubator, **e** experimental set-up

**Fig. 2:**
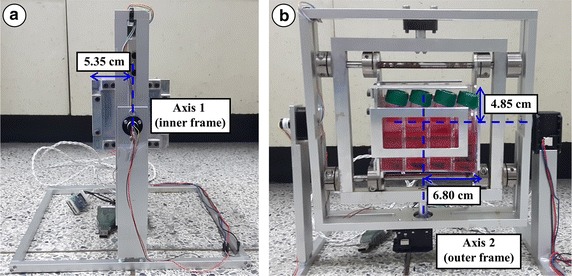
Dimensions of stage for fixing flasks. **a** left side, **b** front side.

**Fig. 3:**
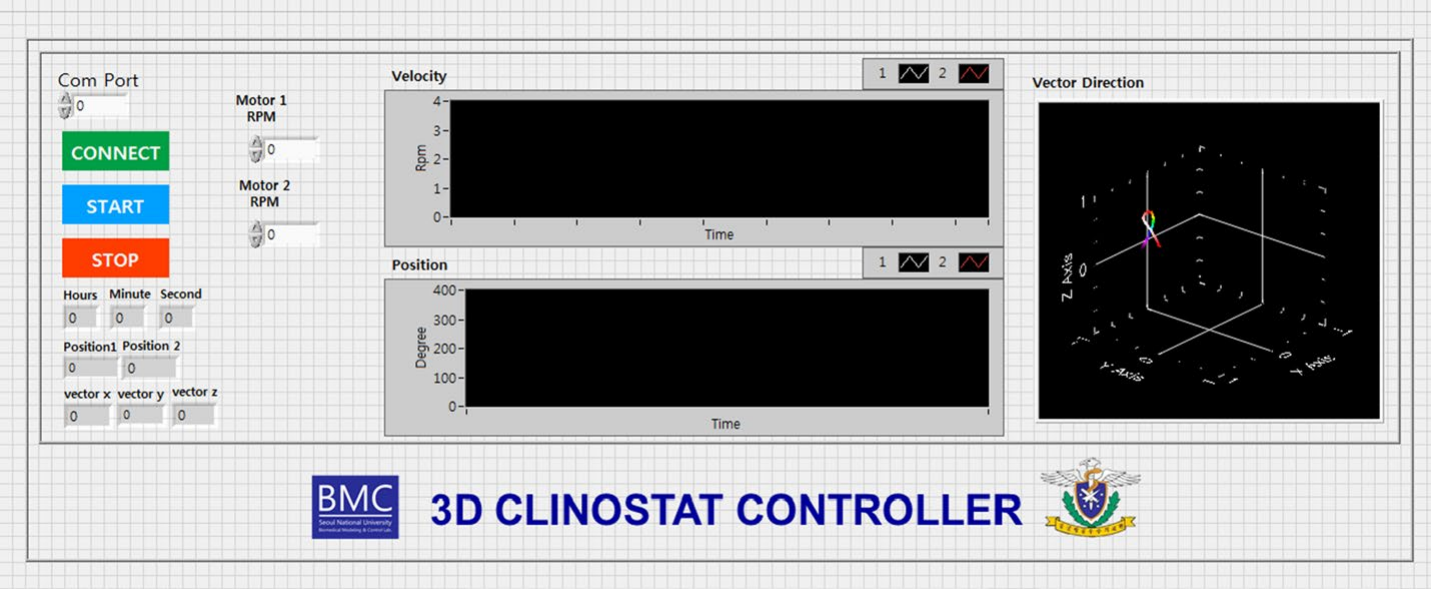
LabVIEW-based graphic user interface for control of 3D clinostat

The 3D clinostat hardware was designed and manufactured according to a conventional 3D clinostat structure described in previous studies (Fig. 1). The clinostat used in this study consists of two perpendicular frames that can rotate independently. The structure of the 3D clinostat was designed using the CAD program (SolidWorks, Dassault Systemes, France, using Seoul National University Academic License) and manufactured using a milling machine with aluminum plates. Two motors (MX-64T, ROBOTIS, Seoul, Republic of Korea) were used to actuate the two frames of the clinostat. Electrical wires for power supply and communication were connected via slip ring, which enables electrical connection between external and rotating frames. The dimensions of the clinostat were limited to 300 × 310 × 350 mm (length × width × height) as the hardware was to be operated within an incubator for cell proliferation. A mechanical stage, which fixes 25 cm^2^ flasks (SPL Life Sciences Co, Korea) for cell cultivation and is capable of accommodating a maximum of eight flasks simultaneously, was affixed at the center of the clinostat (Fig. 1). The quantitative dimensions of the mechanical stage relative to its center of rotation are provided in Fig. 2. The clinostat was controlled by a control algorithm embedded in an external personal computer. The control algorithm provided constant angular velocities for two actuators; the angular velocities were determined by considering the symmetric distribution of the acceleration vector, which consists of the gravitational acceleration and non-gravitational acceleration. The optimal angular velocities were determined according to simulation based on the kinematic model. A graphical user interface was implemented using LabVIEW 2009 (National Instruments, Austin, TX, USA, using Seoul National University Academic License) for convenient and stable operation (Fig. 3).

### Clinostat simulation

**Fig. 4:**
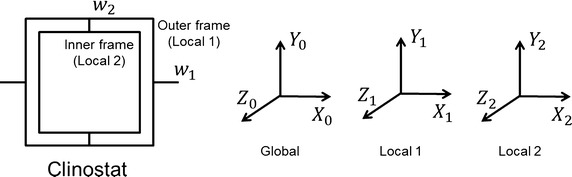
Coordinates of 3D clinostat. Local 1 is the outer frame and Local 2 the inner frame. Global frame refers to the externally observed coordinates

Prior to the cell proliferation experiment, the 3D clinostat should be simulated to guarantee taSMG. One of the most important features of a 3D clinostat is the angular velocities of the two actuators. In order to determine the optimal angular velocity, that enables symmetric acceleration distribution and reduces the effect of non-gravitational acceleration (centrifugal and tangential accelerations), kinematics of the clinostat were modeled, as shown in Eqs. (1)–(9) below (see “List of symbols” at the end of the manuscript). The coordinates are defined as shown in Fig. 4. The global frame indicates the external inertial frame (external observer), the local 1 frame indicates a coordinate used by the outer frame, and the local 2 frame indicates a coordinate used by the inner frame and stage.

1 $$w_{1}^{T}  = [{\dot{\theta }}_{1} ,0,0]{w^{\prime}}_{2}^{T}  = [0,{\dot{\theta }}_{2} ,0]$$

2 $$ w_{2} = R_{x} \left( {\theta_{1} } \right)w^{\prime}_{2} = \left[ {\begin{array}{ccc} 1 & 0 & 0 \\ 0 & {\cos \theta_{1} } & { - \sin \theta_{1} } \\ 0 & {\sin \theta_{1} } & {\cos \theta_{1} } \\ \end{array} } \right]\left[ {\begin{array}{c} 0 \\ {\dot{\theta }_{2} } \\ 0 \\ \end{array} } \right] = \left[ {\begin{array}{c} 0 \\ {\dot{\theta }_{2} \cos \theta_{1} } \\ {\dot{\theta }_{2} \sin \theta_{1} } \\ \end{array} } \right] $$

3 $$ w = w_{1} + w_{2} = \left[ {\begin{array}{*{20}c} {\dot{\theta }_{1} } \\ {\dot{\theta }_{2} \cos \theta_{1} } \\ {\dot{\theta }_{2} \sin \theta_{1} } \\ \end{array} } \right]\;\left( \text{{Summation\;of\;angular\;velocity}} \right) $$

4 $$ \dot{w} = \left[ {\begin{array}{*{20}c} 0 \\ { - \dot{\theta }_{1} \dot{\theta }_{2} \sin \theta_{1} } \\ {\dot{\theta }_{1} \dot{\theta }_{2} \cos \theta_{1} } \\ \end{array} } \right] $$

5 $$ r = R_{x} (\theta_{1} )R_{y} (\theta_{2} ) \left[ {\begin{array}{*{20}c} {\Delta x} \\ {\Delta y} \\ {\Delta z} \\ \end{array} } \right] = \left[ {\begin{array}{*{20}c} {\Delta x\cos \theta_{2} + \Delta z\sin \theta_{2} } \\ {\Delta y\cos \theta_{1} + \Delta x\sin \theta_{1} \sin \theta_{2} - \Delta z\sin \theta_{1} \cos \theta_{2} } \\ {\Delta y\sin \theta_{1} - \Delta x\cos \theta_{1} \sin \theta_{2} + \Delta z\cos \theta_{1} \cos \theta_{2} } \\ \end{array} } \right] $$

6 $$ a\left( t \right) = - \left\{ {\dot{w} \times r + w \times (w \times r)} \right\} $$

7 $$ a(t)^{\prime\prime} = R_{2}^{1} R_{1}^{0} a\left( t \right) = R_{y}^{ - 1} (\theta_{1} )R_{x}^{ - 1} (\theta_{2} )a\left( t \right) = R_{y}^{T} (\theta_{1} )R_{x}^{T} (\theta_{2} )a\left( t \right) $$

8 $$ g(t)^{\prime\prime} = R_{y}^{ - 1} \left( {\theta_{1} } \right)R_{x}^{ - 1} \left( {\theta_{2} } \right)g = R_{y}^{T} \left( {\theta_{1} } \right)R_{x}^{T} \left( {\theta_{2} } \right)g \left( {\because g = \left[ { \begin{array}{*{20}c} 0 & { - 9.8} & 0 \\ \end{array} } \right]^{T} } \right) $$

9 $$ a(t)^{\prime\prime}_{tot} = a(t)^{\prime\prime} + g(t)^{\prime\prime} $$

Among the acceleration experienced by non-inertial frame, local 2, external (gravitational acceleration), centrifugal, and tangential acceleration were considered and Coriolis and radial accelerations were excluded from the analysis. When magnitudes of angular velocities, $\dot{\theta }_{1}$ and $\dot{\theta }_{2}$, are sufficiently small, fluidic movement inside flasks can be negligible. Thus, mechanical condition inside the flasks can be assumed as quasi-static state, and the relative movement inside the flasks is neglected. Therefore, three types of acceleration, centrifugal, tangential and gravitational acceleration, were analyzed in this study.

In order to ensure symmetrical distribution of acceleration, the trajectory of the acceleration vector must avoid asymmetrically distributed path. 3D clinostats with asymmetric acceleration trajectory do not cover all orientations. Therefore, simulation based on the kinematics model was performed using MATLAB R2014b (Mathworks Inc., Natick, MA, USA, using Seoul National University Academic License). Using the Taguchi method [29], which is a well-known optimization approach in mechanical engineering, a combination of two angular velocities was determined from the initial state (1 rpm for angular velocities of outer frame and inner frame). The objective function was diversity of the orientation of the acceleration vectors (including gravitational and non-gravitational acceleration vectors). The magnitude of residual acceleration after 24 h was estimated to guarantee taSMG.

### Cell lines and culture conditions

The human Hodgkin’s lymphoma cell lines L-540 and HDLM-2 were obtained from the German Collection of Microorganisms and Cell Cultures (DSMZ, Braunschweig, Germany) [30]. The human dermal fibroblast (HDF) cells were purchased from the American Type Culture Collection (ATCC, Manassas, VA, USA). L-540 and HDLM-2 cells were maintained in RPMI 1640 (Life Technologies, Gaithersburg, MD, USA) supplemented with 10% fetal bovine serum (FBS, Life Technologies) and 1% penicillin/streptomycin solution (Life Technologies) at 37 °C in 5% CO_2_. HDF cells were maintained in Dulbecco’s Modified Eagle’s Medium (DMEM, HyClone, South Logan, Utah, US) supplemented with 10% FBS and 1% penicillin/streptomycin solution at 37 °C in 5% CO_2_.

### Operating 3D clinostat

Cells were seeded in 25 cm^2^ flasks with the total 5 × 10^6^ cells. Before being placed in the clinostat, the flasks were carefully filled with medium (approximately 80 ml) without air bubbles in order to avoid shearing of the fluid [31]. After the flasks were fixed on the stage of the clinostat, the clinostat was operated for 1, 2, and 3 days in a commercially available incubator set at 37 °C and supplied with 5% CO_2_. The same cells were grown in parallel at 1 G comprised the control culture, which was kept statically in the same incubator as the clinostat. The same procedure was repeated for four times.

### Cell proliferation assay

Cell counting was performed manually with a hemocytometer (Biosystems, Nunningen, Switzerland) on trypan blue (Life Technologies) treated cells to assess cell concentration and viability, according to the dye exclusion method. The counts were carried out in triplicate per independent sample.

### Statistical analysis

Cell counting data were represented as means with standard error of the mean (SEM). Statistical significance was determined via a two-tailed Student’s t test and analyzed using Graph Pad Prism 6 (Graph Pad Software, Inc., San Diego, CA, USA). Differences were considered to be significant at *p* < 0.05.

## Results

### 3D clinostat simulation

**Fig. 5:**
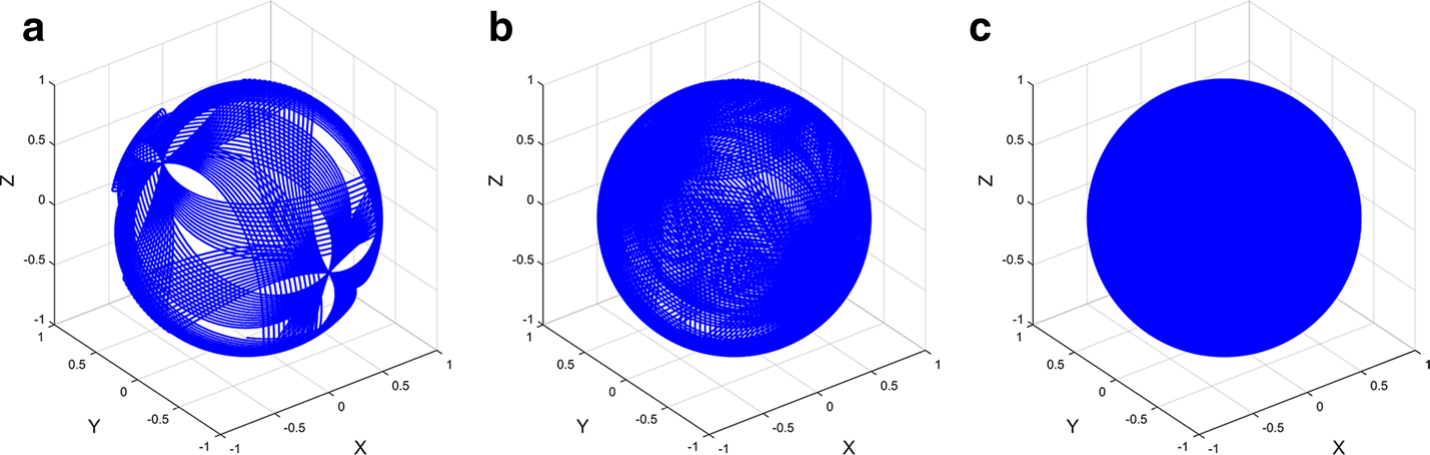
Acceleration distribution of 3D clinostat with optimized angular velocities (outer frame: 0.913 rpm, inner frame: 0.683 rpm); acceleration distribution after **a** 1 h, **b** 2 h, and **c** 3 h

**Fig. 6:**
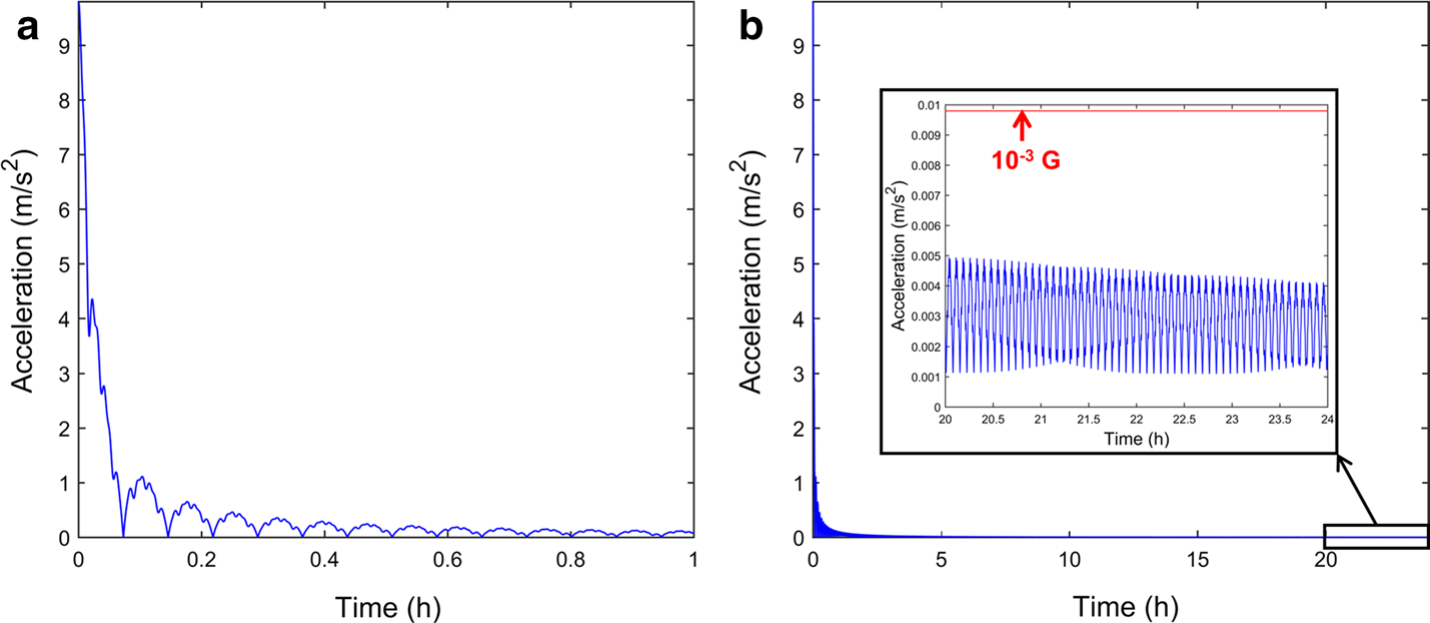
**a** Time-averaged acceleration from 0 to 1 h, **b** Time-averaged acceleration from 0 to 24 h. The location of analysis is [Δx Δy Δz] = [0.1 m 0.1 m 0.1 m] from the center of the clinostat. The acceleration is smaller than 10^−3^ G when reached 24 h

**Fig. 7:**
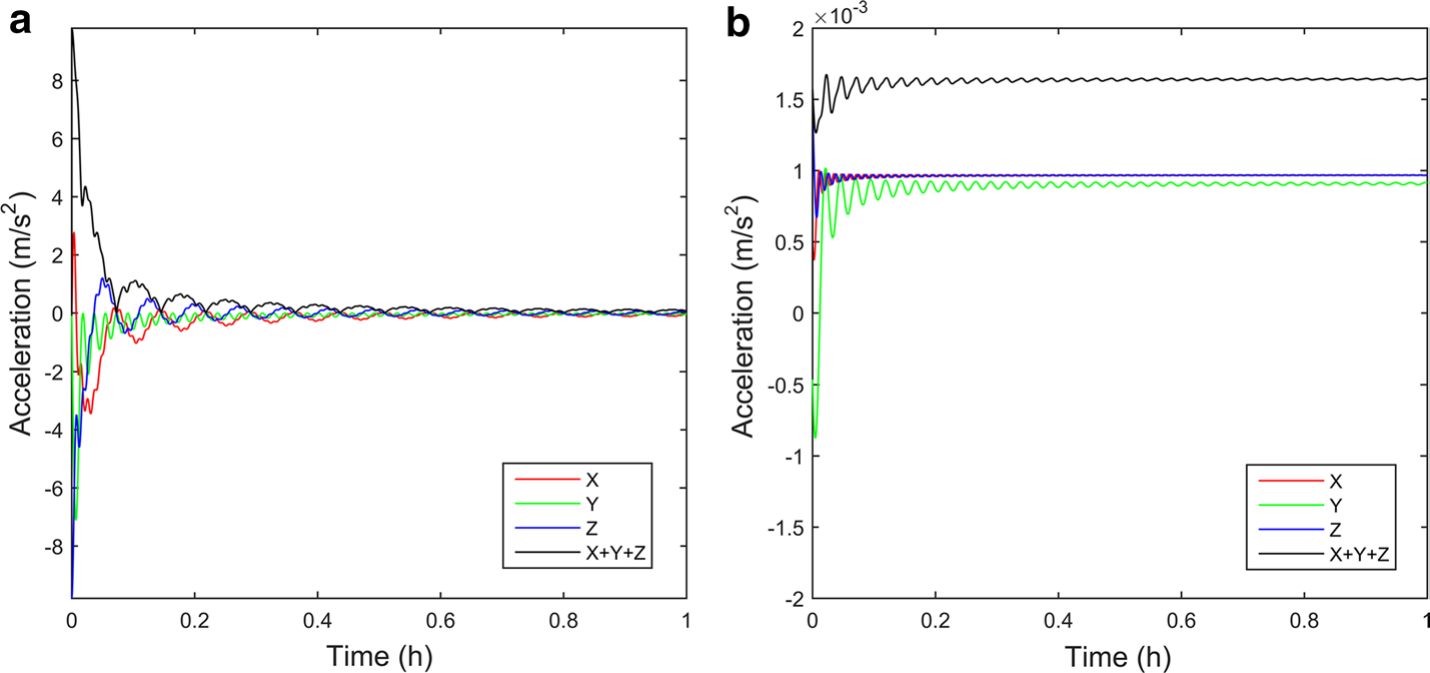
Time-averaged gravitational and non-gravitational acceleration. The acceleration consists of **a** gravitational acceleration and **b** non-gravitational acceleration. The gravitational acceleration decreases continuously whereas the non-gravitational acceleration does not asymptote to zero

The optimization approach, using the Taguchi method, enabled angular velocities of the outer and inner frame to reach 0.913 and 0.683 rpm, respectively. The angular velocities are less than 5.5°/s and the mechanical condition inside rotating flasks can be approximated to quasi-static state. Thus, it can be assumed that mechanical condition inside rotating flasks is similar to that of stationary flasks. The combination of the two angular velocities showed symmetric acceleration distribution, as shown in Fig. 5. Using the two optimized angular velocities, simulation of microgravity using the 3D clinostat was performed, and acceleration for 24 h was calculated for validation. A point with relative position [Δx Δy Δz] = [0.1 m 0.1 m 0.1 m] measured from the center of the clinostat was assumed. Assumed relative position is further away center of rotation more than any other points of the flasks fixed in stage. Since residual acceleration, which interrupts nullification of gravity, increases as distance from center of rotation becomes farther, the position [0.1 m 0.1 m 0.1 m] is appropriate for reliable simulation. The gravitational, centrifugal, and tangential accelerations were considered in calculation of time-averaged acceleration, which indicates taSMG, as shown in Fig. 6. As shown in figure, time-averaged acceleration decreases as damped oscillation for 1 h. After 24 h, the time-averaged acceleration, which consists of the gravitational and non-gravitational acceleration vectors, enabled the 3D clinostat to provide the condition of 1.7 × 10^−4^ G. The time-averaged gravitational acceleration also decreased with damped oscillation, as shown in Fig. 7a, and reached 3.3 × 10^−4^ G after 24 h. The time-averaged gravitational acceleration decreased and asymptoted to zero with time; however, the time-averaged non-gravitational acceleration did not demonstrate a decreasing tendency and was relatively constant (Fig. 7b). After 24 h, time-averaged non-gravitational acceleration reached 1.7 × 10^−4^ G, which is almost the same value as that of the initial state.

### Cell proliferation: taSMG inhibits the cell proliferation in human lymphoma cell lines

**Fig. 8:**
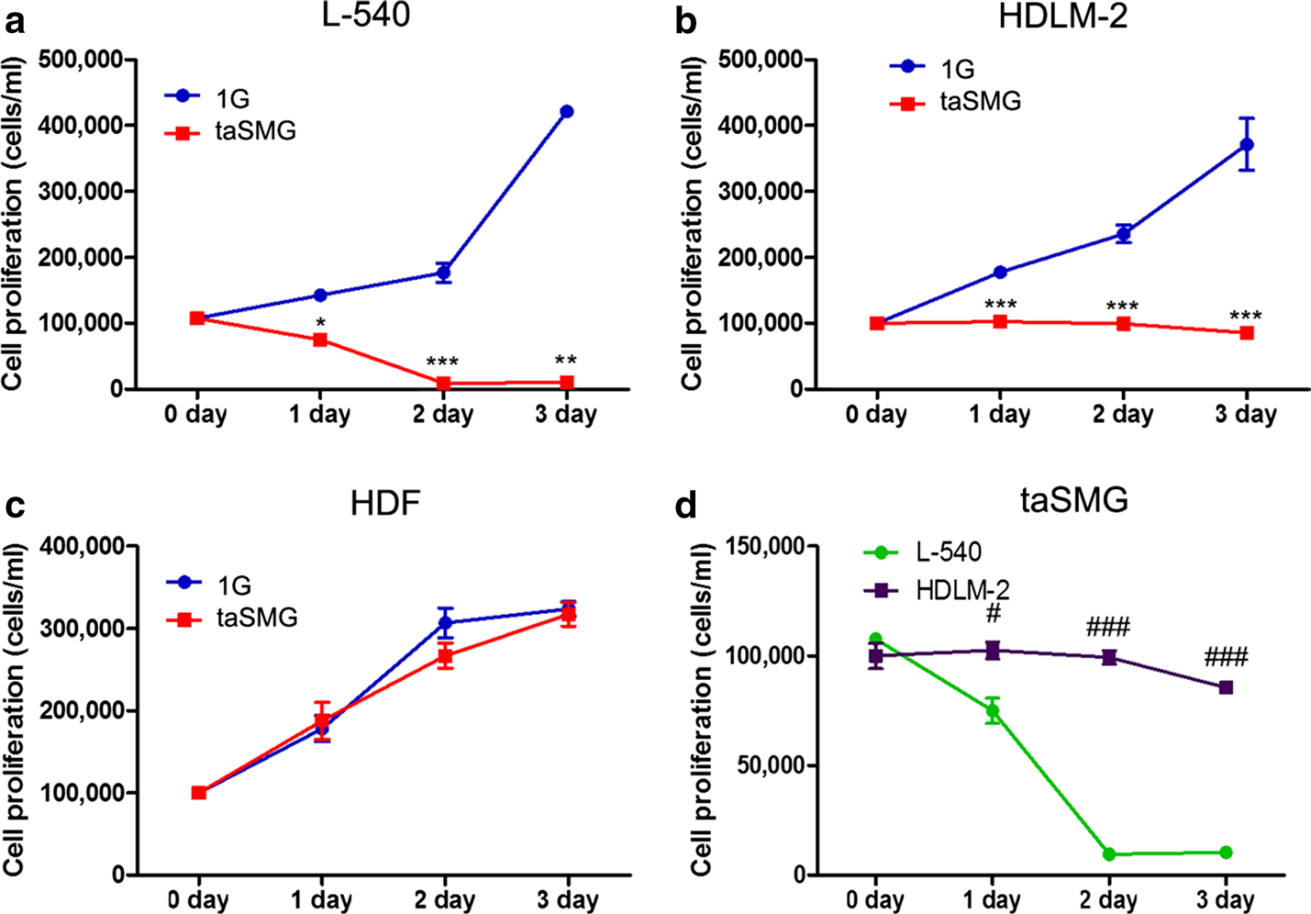
Effect of time-averaged simulated microgravity (taSMG) on cell proliferation. Human lymphoma cell lines, **a** L-540 and **b** HDLM-2, and **c** human dermal fibroblast (HDF) cells were cultured under 1 G or taSMG from 1 to 3 days. **d** Cell proliferation of L-540 cell was compared with that of HDLM-2 cells under taSMG. Data are presented as mean ± SEM; *p < 0.05, **p < 0.005, and ***p < 0.0005 vs. the 1 G control group. ^#^ *p* < 0.05 and ^###^ *p* < 0.0001 vs. the L-540 group

taSMG is known to affect various cellular processes [25–28]. In order to investigate whether taSMG elicits varying physiological changes in tumor cells relative to normal cells, we examined the cell viability of human lymphoma cells (L-540 and HDLM-2 cells) and normal HDF cells regulated under taSMG. After cell culture under taSMG for 1, 2, and 3 days, cell proliferation was analyzed. The taSMG was found to inhibit the proliferation of L-540 and HDLM-2 cells, but did not affect the growth of HDF cells (Fig. 8a–c). These data indicate that taSMG selectively inhibits cell growth in human lymphoma cells, but not in normal cells. Additionally, when L-540 and HDLM-2 cells under taSMG were compared, proliferation of L-540 cells was inhibited to a larger extent than that of HDLM-2 cells (Fig. 8d).

## Discussion

The present 3D clinostat with optimized angular velocities was tested for its nullification of gravity vector without repeated trajectory using kinematics model based simulation. The time-averaged acceleration was gradually decreased, and reached 1.7 × 10^−4^ G after 24 h of operation. Compared with previous studies [22], the present results indicate that sufficient time-averaged acceleration was provided with symmetric acceleration distribution. The time-averaged acceleration did not asymptote to zero-gravity as centrifugal acceleration was applied to radial direction continuously and therefore not cancelled. However, the centrifugal acceleration is proportional to the square of the angular velocity, and the angular velocities provided in this study were sufficiently small (0.913 and 0.683 rpm). In order to minimize the residual acceleration of the 3D clinostat and assume quasi-static state, operation with minimum angular velocities is required. However, this may result in the taSMG generated not being recognized by cells, as a consequence of the extremely slow change in orientation.

Numerous in vitro studies have shown that taSMG affects various cellular processes. In the present study, we investigated whether exposure to taSMG, provided by 3D clinostat, alters cell proliferation. Unexpectedly, lymphoma cell lines L-540 and HDLM-2 showed inhibition of cell growth whereas the growth of HDF cells was not affected under taSMG (Fig. 8). In addition, it was found that taSMG induces cell death of lymphomas. Our data indicate that further investigation of the specific mechanisms underlying taSMG-induced cell death in lymphoma cells is required. Further, additional experiments with different physical condition such as angular velocities and cell concentration should be performed. Previous studies [25, 32] have reported cell viability of almost 100% under taSMG, with only cell proliferation inhibited. In contrast, the cell death of both L-540 and HDLM-2 occurred in the current study. These contrasting results may be due to differences in physical condition between our study and the previously reported studies, making it difficult to compare the data. Therefore, for improved comparability of results, the alteration of relative cell proliferation and viability should be investigated under various angular velocities and cell concentration.

Even though results show that taSMG inhibits proliferation of lymphoma, L-540 and HDLM-2, it has still limitations. First, taSMG is actually a different physical condition compared to real microgravity. Thus, it is hard to draw a conclusion that the cancer cells are inhibited in the real microgravity condition. Rather, it is more meaningful to disclose biological mechanism by investigating signalling pathway how cancer cell respond to the stress of taSMG. It is expected that the mechanism can contribute to development of novel biological and chemical approach to treat cancer. Second, the cancer cells used in this study were in free floating whereas HDF cells adhere on the wall of the flask. Thus, the physical condition experienced by the cancer and HDF cells are quite different. Even though the angular velocity was controlled to be sufficiently small and quasi-static state is assumed, method of experiment should be more improved to minimize the physical difference between two groups. Utilizing cancer cell of adherent type can be considered for alternative of using L-540 and HDLM-2. Ultimately, it must be verified experimentally in real microgravity condition of space.

Recently, the necessity for simulated partial microgravity [21], to provide a Mars (0.38 G) or moon (0.17 G) like environment, was suggested. An additional study was performed to compare three types of partial gravity algorithms by applying them to mouse skeletal myoblasts and human lymphocytes [32]. This study showed that exposure to partial gravity affects cell proliferation, and these effects vary according to the type of algorithm applied. Accordingly in future studies, implementation of a partial microgravity algorithm and its application to proliferation of various types of cell can be executed for support of future planetary exploration programs.

## Conclusions

A 3D clinostat, suitable for operation in a conventional incubator, was designed and manufactured. Additionally, optimized angular velocities for symmetric acceleration distribution were determined using an optimization approach, namely the Taguchi method. The simulation results tested that the 3D clinostat could provide proper taSMG without repeated trajectory of acceleration vector. Using the 3D clinostat, it was demonstrated that the proliferation of human lymphoma cells was significantly inhibited under taSMG, whereas that of HDF cells was unaffected. Findings in this research are of potential value for the development of candidate therapies against lymphoma.

## Abbreviations

taSMG: time-averaged simulated microgravity; HDF: human dermal fibroblast.

### List of symbols

$$ w_{1}$$
angular velocity of the outer frame with respect to the global frame
$$ w_{2} $$
angular velocity of the inner frame with respect to the global frame
$$ w^{\prime}_{2} $$
angular velocity of the inner frame respect to Local 1 frame
$$ {\text{w}} $$
summed angular velocity vector with respect to the global frame
$$ \Delta x,\Delta y,\Delta z $$
position deviation from clinostat center with respect to Local 2 frame
$$ {\text{r}} $$
position deviation from clinostat center with respect to the global frame
$$ a(t) $$
non-gravitational acceleration of stage with respect to the global frame
$$ a(t)^{\prime\prime} $$
non-gravitational acceleration of stage with respect to Local 2 frame
$$ g(t)^{\prime\prime} $$
gravitational acceleration with respect to Local 2 frame
$$ g(t) $$
gravitational acceleration with respect to the global frame
$$ a(t)^{\prime\prime}_{tot} $$
acceleration with respect to Local 2 frame
$$ R_{x} , R_{y} $$
rotational matrix with x and y axes, respectively
